# The Presence of the Endocannabinoid System in an In Vitro Model of Gorham-Stout Disease and Its Possible Role in the Pathogenesis

**DOI:** 10.3390/ijms26031143

**Published:** 2025-01-28

**Authors:** Cinzia Aurilia, Gaia Palmini, Simone Donati, Irene Falsetti, Gianna Galli, Lorenzo Margheriti, Teresa Iantomasi, Arcangelo Moro, Maria Luisa Brandi

**Affiliations:** 1 Department of Experimental and Clinical Biomedical Sciences, University of Florence, Viale Pieraccini 6, 50139 Florence, Italy; cinzia.aurilia@unifi.it (C.A.); simone.donati@unifi.it (S.D.); irene.falsetti@unifi.it (I.F.); gianna.galli@unifi.it (G.G.); teresa.iantomasi@unifi.it (T.I.); 2 Fondazione Italiana Ricerca sulle Malattie dell’Osso (FIRMO Onlus), 50141 Florence, Italy; gaia@fondazionefirmo.com; 3 Stabilimento Chimico Farmaceutico Militare (SCFM)—Agenzia Industrie Difesa (AID), 50141 Florence, Italy; lorenzo.margheriti@aid.difesa.it (L.M.); arcangelo.moro@aid.difesa.it (A.M.)

**Keywords:** GSD, ES, hBMMSCs, osteogenic differentiation

## Abstract

Gorham-Stout syndrome (GSD), also known as disappearing bone disease, is an extremely rare bone disorder, characterized by a huge bone loss, which is followed by a lack of new matrix deposition and an excessive proliferation of both blood vessels and lymphatics. Unfortunately, the biological causes of GSD are still unknown. Recent studies that have tried to understand the etiopathogenesis of GSD have been principally focused on the vascular and osteoclastogenic aspects, not considering the possibility of a lack of osteoblast function. Nowadays, a diagnosis is still difficult, and is often made by exclusion of the presence of other pathologies, as well as on radiological evidence, and finally confirmed by histological examination. Treatment also remains a critical issue for clinicians today, who mostly try to control the progression of the disease. Over the last two decades, clear evidence has emerged that the endocannabinoid system plays an important role in bone metabolism, leading scientists to hypothesize that it could be involved in physiological and pathological bone processes. In this work, we analyzed the presence of the ES in a primary cell line of human mesenchymal stem cells derived from a GSD patient for the first time, to understand if and how this complex network may play a role in the pathogenesis of the syndrome. Our preliminary results demonstrated that the ES is also present in the pathological tissue. Moreover, the qRT-PCR analysis showed an altered expression of the different ES components (i.e., CNR1, CNR2, TRPV1, and GPR55). We observed an upregulation of CNR1 and TRPV1 expression, while the opposite trend was noticed for CNR2 and GPR55 expression. Thus, these results could lead us to speculate that possible deregulation of the ES may play an important role in the lack of bone regeneration in GSD patients. However, further studies will be necessary to confirm the role of the ES in the progression of GSD and understand whether the natural components of Cannabis Sativa could play a therapeutic role in the treatment of the disease.

## 1. Introduction

Gorham-Stout disease (GSD), or “vanishing bone syndrome”, is an extremely rare skeletal disorder, characterized by the onset and progression of a massive and conspicuous osteolysis, followed by a lack of new bone tissue deposition [1]. Although J.B.S. Jackson discovered the syndrome in 1838, only in 1955 did L.W. Gorham and A.P. Stout describe the main characteristics of GSD (i.e., lymphangiomatosis and osteolysis) [2].

At present, the prevalence of GSD is around 350 cases worldwide; however, the scientific community agrees that its incidence may be higher since it is believed to be caused by misdiagnosis or even non-diagnosis of the pathological condition. However, despite the fact that there seems to be a higher prevalence of occurrence in young adults, no gender, age, or race preference have been observed [3].

Generally, all bones of the body can be affected by the process of osteolysis, but a greater involvement of the upper part of the skeleton has been seen, with the pelvic, skull, rib, clavicle, and vertebrae bones being particularly affected [1]. Based on the bone districts affected and the areas of bone affected by osteolysis, patients may experience different clinical courses. In fact, the involvement of bones that protect vital organs, such as the spinal column and chest, could lead to the development of life-threatening complications for the patient, especially if not treated in time. It has been observed that imaging examinations (i.e., X-rays, computed tomography, 99 mTc(V)-DMSA, etc.) can represent an important diagnostic strategy for GSD. In addition to these instrumental tests, biochemical tests can be used to assess the blood levels of various markers of bone turnover, inflammation, and angiogenesis, which are often increased in GSD patients compared to the healthy population. However, a diagnosis of GSD is almost always made by excluding the presence of oncological, infectious, or endocrine disorders in the patient, to be subsequently confirmed by histological analysis [3,4,5,6,7,8,9,10,11].

The treatment of this disease is also not easy. In fact, being an extremely rare disease, guidelines for its management have never been drawn up and the available therapies focus more on controlling progression, thus involving radiotherapy, anti-angiogenic drugs, and surgery [1].

The endocannabinoid system (ES) is a complex network of ligands (i.e., 2-Arachidonoylglycerol (2-AG) and Anandamide (AEA)), receptors (i.e., Cannabinoid Receptor type 1 (CNR1), type 2 (CNR2)), and enzymes responsible for the synthesis and degradation of 2_AG (i.e., diacylglycerol lipases (DAGLα/β) and monoacylglycerol lipase (MAGL)) and the synthesis and degradation of AEA (i.e., Nacyl-phosphatidylethanolamines-specific phospholipase D (NAPE-PLD) and fatty acid amide hydrolase (FAAH)). Recent studies have reported that ES is involved in different physiological mammalian mechanisms [12], including bone remodeling [13].

The latter is defined as a continuous process of bone matrix resorption and deposition that enables the maintenance of skeletal structural integrity and normal calcium and phosphorus levels in the body. The cells involved in this process are osteoclasts (OCs) and osteoblasts (OBs), whose altered balance in terms of number and function leads to the development of skeletal diseases [14].

Knowledge about the role of ES in bone metabolism is quite young. Nevertheless, the discovery of the presence of ES components in OBs and OCs has led the scientific community to focus on understanding how this molecular machinery may play a role in bone turnover.

The first study was conducted by Ofek et al. [15], in 2005, who showed not only that CNR1 and CNR2 were present in both OBs and OCs, but that activation of CNR2 was responsible for reduced osteoclastogenesis and increased OB formation and differentiation. This finding paved the way for several research works that highlighted that CNR2 would appear to have a protective action on bone tissue, while CNR1 may have an age-dependent effect on bone mass by regulating the differentiation of OCs and OBs [16,17,18,19,20].

In addition to the classical ES receptors, it has been seen that OCs and OBs also express the G protein-coupled receptor 55 (GPR55) and the transient receptor potential cation channel subfamily V member 1 (TRPV1), which are targets of both synthetic cannabinoids and endocannabinoids [21]. Although GPR55 is considered the third cannabinoid receptor, few studies have investigated its possible role in bone metabolism and have mainly focused on its ability to modulate OCs’ differentiation and activity [17]. Instead, studies on TRPV1 have shown that stimulation of this receptor positively affects OCs’ function and negatively affects OBs’ function, making them, along with CNR2, a promising target in the treatment of skeletal pathological states [22].

In relation to all of the above aspects, the principal aims of this study have been to establish a primary cell line of human bone marrow-derived mesenchymal stem cells (hBMMSCs) from an osteolytic lesion of a GSD patient and to evaluate the presence of ES and how this network could be involved in the syndrome’s pathogenesis. So, in this work, we demonstrated the presence and a deregulation of the ES components in hBMMSCs derived from a GSD patient for the first time, paving the way to the hypothesis that the ES could be involved in the onset and progression of GSD, leading to the possibility of developing future therapies based on the use of the non-psychotropic natural components of *Cannabis Sativa*.

## 2. Results

### 2.1. Isolation and Characterization of hBMMSCs from GSD Patient

**Figure 1:**
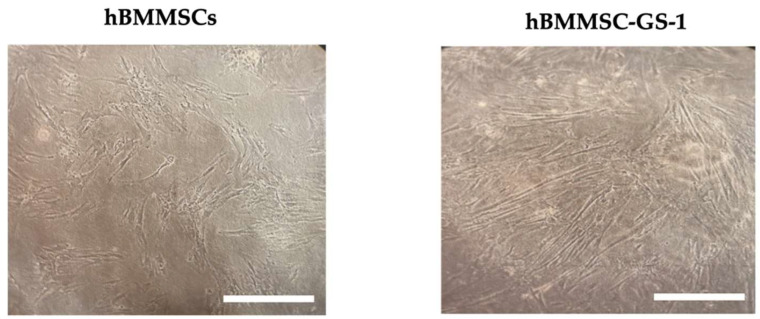
Representative observation of primary hBMMSCs derived from bioptic samples from GSD patient (B) and of primary hBMMSC line. Observation in phase contrast. Original magnification 10×. Scale bar: 200 μm.

A primary cell line was established from a biopsy sample of bone marrow from an osteolytic lesion of a GSD patient, marked as hBMMSC-GS-1. The established cell line displayed a characteristic spindle-shaped morphology similar to that of the healthy hBMMSCs (Figure 1).

The expanded cells were subsequently characterized by immunofluorescence staining to verify their phenotype, analyzing the presence of the mesenchymal stem cell markers (i.e., CD105, CD90, and CD44) and one of the hematopoietic markers (CD34).

**Figure 2:**
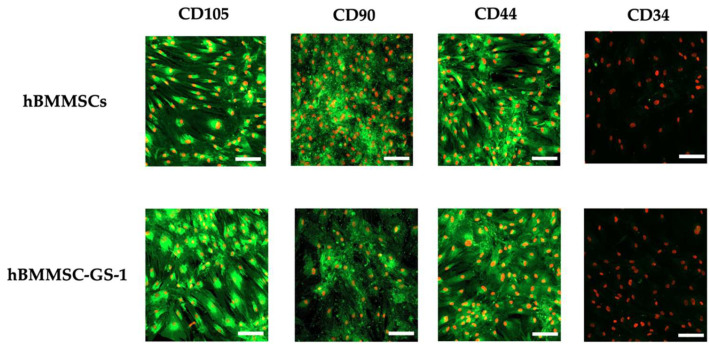
Immunofluorescence staining of CD105, CD90, CD44, and CD34 in hBMMSC-GS-1 line vs. hBMSC cell lines. Nuclei were counterstained with propidium iodide. LSCM in conventional colors: green for markers and red for nuclei. Original magnifications: 10×. Scale bar: 100 µm.

Immunofluorescence staining, observed in LSCM, showed the presence of all of the analyzed mesenchymal stem cell markers and the absence of CD34, as reported for healthy hBMMSCs, confirming their mesenchymal phenotype (Figure 2).

### 2.2. Adipogenic and Osteogenic Differentiation Potential of hBMMSC-GS-1 Line

The evaluation of the possible alteration of the multipotency of the hBMMSC-GS-1 line with respect to a healthy hBMMSC line was assessed by the induction toward the adipogenic and osteogenic phenotype.

**Figure 3:**
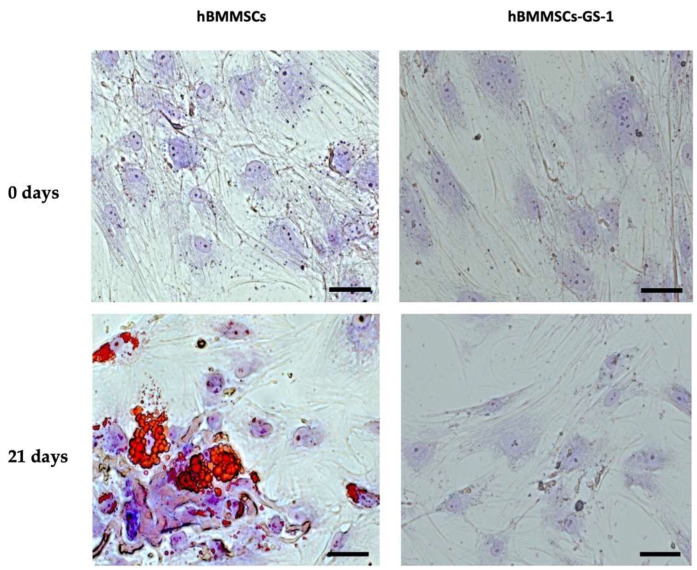
Adipogenic differentiation assay: representative images of adipogenic phenotype evaluation of hBMMSC-GS-1 and HBMMSC lines at 0 and 21 days of induction. The intracellular lipidic vesicles are stained in red by Oil Red O staining, while nuclei were counterstained with toluidine blue and are visible in blue/violet. Images acquired through brightfield microscopy. Original magnifications: 10×. Scale bar: 100 µm.

The subsequent adipogenic differentiation assay showed that, although both hBMMSC-GS-1 and hBMMSCs were cultured in AM, only hBMMSCs, after 21 days of induction, using Oil Red O staining were able to differentiate showing an accumulation of multiple intracellular lipid-filled vesicles compared to BMSC-GS-1, which showed an altered differentiation capacity (Figure 3).

As reported for the adipogenic differentiation assay, the osteogenic differentiation assay was also observed with a different capacity to differentiate in the analyzed cell lines. In fact, only the hBMMSC line showed the ability to differentiate into osteoblasts after 14 days of induction, forming mineralized HA deposits after 35 days of induction, whereas this capacity was lacking for the hBMMSC-GS-1 cell line.

**Figure 4:**
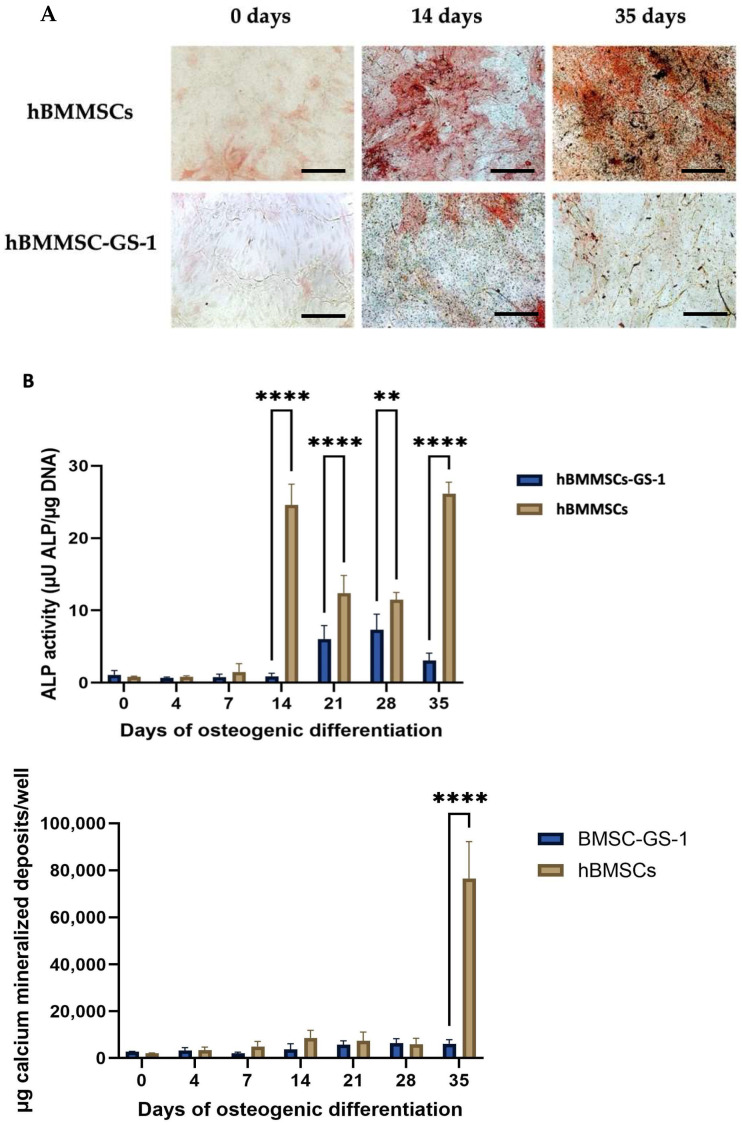
Osteogenic differentiation assays. (**A**) Representative images of ALP and HA cytochemical staining performed on hBMMSc and hBMMSC-GS1 lines at 0, 14, and 35 days of induction. The ALP positive cells are in red, while the HA deposits are stained in black. Nuclei were counterstained with methyl green. The images were acquired through brightfield microscopy. Original magnifications: 20×. Scale bar: 100 µm. (**B**) Quantitative analyses of ALP enzymatic activity (up top) and HA mineralized deposits (top down) in hBMMScs and hBMMSCs-GS-1 cultured in OM up to 35 days. Experiments were carried out in quadruplicates in three independent experiments (*n* = 12) and results are mean ± SD. ** *p* < 0.01 versus hBMMSCs; **** *p* < 0.0001 versus hBMMSCs.

Also, the ALP activity and HA deposits’ spectrofluorimetric assay revealed the failure of BMSC-GSD-1 cells to differentiate in an osteogenic sense (Figure 4).

This could signify that the lack of bone tissue regeneration in GSD may be due to the incapacity of BMSCs to differentiate in bone cells, showing that the excessive resorption of bone tissue, followed by non-deposition of a new bone matrix, leads to the formation of the characteristic osteolytic gaps found in patients with the syndrome.

### 2.3. Expression Profile of ES Components

**Figure 5:**
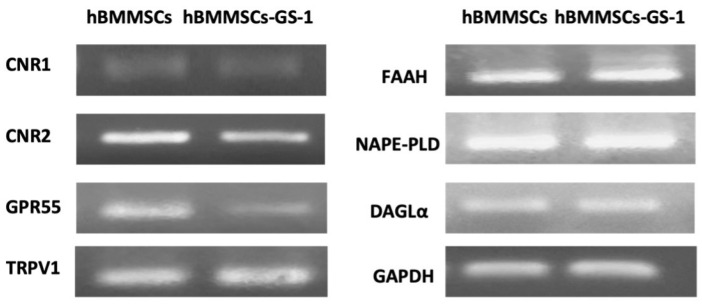
Gene expression of ES components (i.e., *CNR1*, *CNR2*, *GPR55*, *TRPV1*, *FAAH*, *NAPE-PLD*, and *DAGL*α) in hBMMSC and hBMMSC-GS-1 lines. *GAPDH* was used as a housekeeping gene.

We performed qualitative RT-PCR which assessed the presence of analyzed ES components (listed in the Materials and Methods section), revealing a low expression of the *CNR1* gene in both analyzed cell lines and a lower expression of *CNR2* and *GPR55* in the hBMMSC-GS-1 cell line compared to the healthy control. On the contrary, the *TRPV1* receptor gene would seem to be more expressed in the hBMMSC-GS-1 line than in the hBMMSC line, while the *FAAH*, *NAPE-PLD*, and *DAGL* enzymes seemed to be equally expressed in both cell lines (Figure 5).

**Figure 6:**
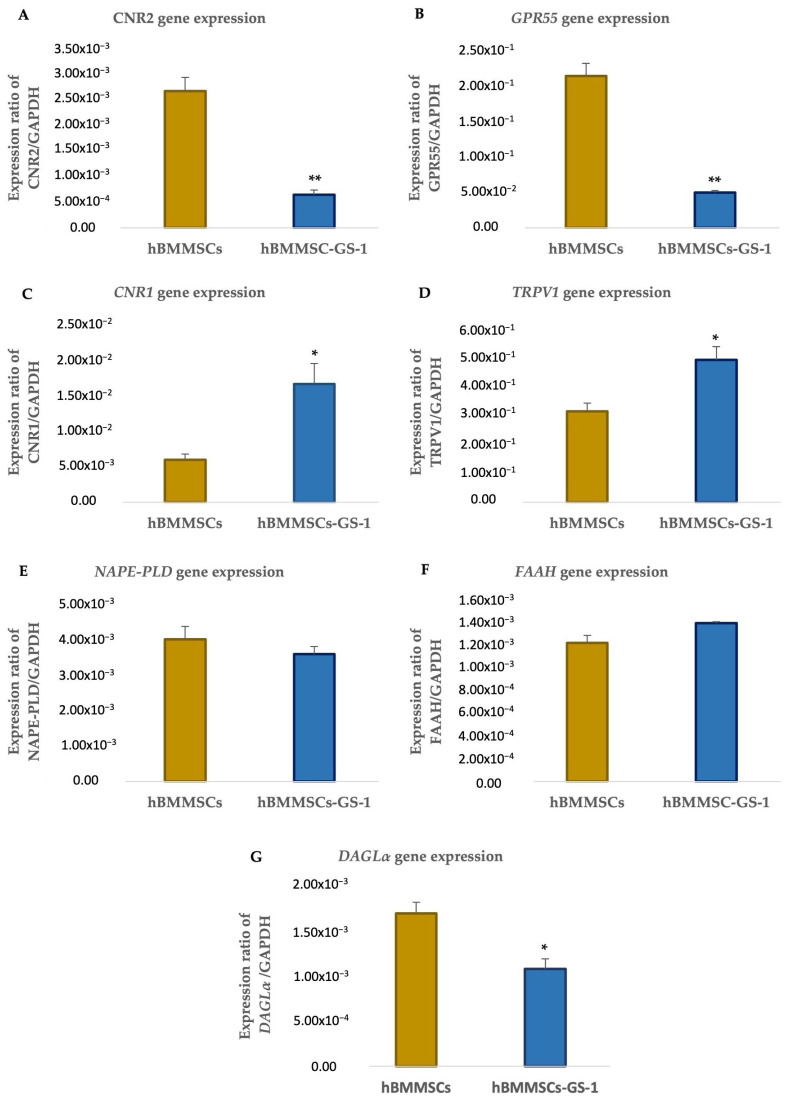
Analysis of gene expression of *CNR2* (**A**), *GPR55* (**B**), *CNR1* (**C**), *TRPV1* (**D**), *NAPE-PLD* (**E**), *FAAH* (**F**), and *DAGLα* (**G**) in hBMMSC and hBMMSC-GS-1 cells. Analysis was performed on cells cultured in GM. Experiments were carried out in triplicate for each gene in three independent experiments (*n* = 9). Values are expressed as mean ± SD and were normalized to the housekeeping gene *GAPDH*. * *p* < 0.05 versus hBMMSCs; ** *p* < 0.01 versus hBMMSCs.

Subsequent qRT- PCR evaluation confirmed a lower expression of *CNR2* and *GPR55* in the hBMMSC-GS-1 line compared to the healthy control (Figure 6A,B). On the contrary, the *CNR1* and *TRPV1* receptors were more expressed in the hBMMSC-GS-1 line with respect to hBMMSCs (Figure 6C,D). No difference in expression levels was reported for the *NAPE-PLD* and *FAAH* genes (Figure 6E,F), while a difference was reported for *DAGL*α, which was less expressed in the hBMMSC-GS-1 line than in the hBMMSC line (Figure 6G).

## 3. Discussion

Nowadays, despite several studies being conducted on GSD, the cellular and molecular mechanisms underlying the onset and development of this pathology are still poorly understood, making the diagnosis and treatment still critical [1].

Two scientific theories have been present to date regarding the pathogenesis of GSD, which are principally focused on possible abnormalities of the vascular and lymphatic systems rather than a failure in the bone tissue regenerating process [23,24].

In recent years, the role of ES in the bone mineralization process has been demonstrated [13] and has paved the way for the future development of new therapeutic approaches for bone diseases. Indeed, it is clear from the various preclinical studies that both the modulation of CNR1 at the presynaptic and bone cell level and the activation of CNR2 at the osteoblastic level can play a very important role in bone turnover. The mechanism through which CNR1 can regulate bone metabolism has yet to be fully elucidated, given the few studies present in the literature, which have also reported conflicting data. Indeed, in vivo studies have shown that CD1CB1−/− rats had increased bone mass compared to C57CB1−/− rats, which had reduced bone mass compared to the control [25,26]. However, the positive effect of a CNR1 deficiency on osteoblastogenesis occurred only in the early stages of bone development, whereas accelerated osteoporosis arose in animals during adulthood, probably due to a shift in bone marrow cell differentiation towards the adipocyte lineage at the expense of the osteoblastic lineage [18,27]. However, a lower expression of CNR1 than CNR2 in bone cells would suggest a lower influence of the first on bone metabolism. In addition, the effect of CNR1 would be more pronounced at the peripheral sympathetic nerve terminals, where its activation would inhibit the release of norepinephrine, resulting in increased osteoblast differentiation and activity and reduced OC formation [26,28,29,30]. All of this evidence tells us that CNR1 plays an important role in the formation of normal bone tissue, but its involvement in bone metabolism is very complex.

Also, studies conducted on the role of CNR2 in bone turnover have produced conflicting results. However, accumulating scientific evidence has attributed a protective role to CNR2 regarding bone tissue. Preclinical studies in animal models have not only shown that CNR2 deficiency was responsible for the reduced number and function of OBs and increased number of OCs, but also that this deficiency caused changes exclusively in bone tissue, without affecting any other organ [31,32,33,34]. Moreover, it has also been demonstrated that CNR2 activation in hFOB 1.19 cells was able to increase their ALP activity and the production of a mineralized bone matrix, and that polymorphisms of the gene encoding for CNR2 are highly correlated with the occurrence of osteoporosis [35,36].

In light of this, and according to the pathophysiology of the syndrome, we have shown, firstly, that hBMMSC-GS-1 cells are unable to differentiate into osteoblasts, exhibiting a reduction in both ALP levels and the production of hydroxyapatite crystal deposits when compared to healthy hBMMSCs. After this, we observed that CNR1 expression is higher in BMSCs derived from GSD patients than in control cells, while the opposite trend has been observed for CNR2. Given the few and controversial data found in the literature about the role of CNR1 in bone remodeling, it is difficult to say whether the upregulation of CNR1 gene expression in our GSD cellular model can negatively regulate its osteoblastic differentiation process and further studies are needed to clarify this aspect.

In addition to the canonical SE receptors, we also evaluated the possible deregulation of the CB-like receptor TRPV1 gene and the orphan receptor, GPR55, since Rossi et al. [37] showed not only that TRPV1 is expressed in human OBs together with CNR2, but also that the use of a selective TRPV1 agonist and CNR2 antagonist led to reduced osteoblast differentiation, whereas the opposite effect was obtained by CNR2 stimulation [37]. Moreover, they also showed that TRPV1 activation on OCs increased the number of Tartrate-Resistant Acid Phosphatase (TRAP)-positive cells, with an increase in both TRAP gene and cathepsin K expression [38]. In addition, mice deficient in the TRPV1 gene have been shown to have increased bone mass, probably because osteoclast precursors in these animals are less sensitive to stimulation by RANK-L, reinforcing the idea that TRPV1 should be considered a potential target for preventing bone diseases [22].

Firstly, the data obtained in our study confirmed the expression of TRPV1 in the healthy hBMMSC line, but we also observed that the expression of this receptor is higher in an hBMMSC-GS-1 line than in a healthy line. As previously reported, CNR2 stimulation leads to an increase in bone matrix formation, while stimulation of TRPV1 inhibits osteogenesis [37]. Therefore, the results that we observed about the inverted expression levels of these two genes in hBMMSCs-GS-1 with respect to control cells are really interesting and should be better investigated to understand whether this mechanism may contribute to the failure of bone regeneration in GSD patients.

In association with the reduced expression of CNR2, GPR55 also showed the same trend in GSD patient cells compared to control cells. Although this receptor is considered one of the orphan receptors responsive to cannabinoids, and its expression was found in both OCs and OBs, its potential role in bone remodeling is still not entirely clear. The few works conducted on this topic provide us with more data about the action of GPR55 on OC activity and differentiation [17]. Indeed, both in vitro and in vivo studies have shown that activation of this receptor decreased OC formation but increased their resorptive activity, while the opposite was achieved with inactivation or deletion of GPR55 [39]. Furthermore, GPR55−/− animals did not show any abnormalities in the proliferation and activity of OBs, and the use of the GPR55 agonist on osteoblastic cells did not significantly influence their bone matrix production [39]. Although it would appear that this receptor has no action on bone formation, it has been seen that GPR55 inhibition and CNR2 activation by cannabidiol (CBD) promoted MSCs’ migration, via p42/44 MAPK activation, and that the long-term exposure of MSCs to CBD increased the differentiation of these cells in an osteogenic sense, revealing the potential osteoanabolic properties of CBD [40].

Our results show that GPR55 is less expressed in the hBMMSC-GS-1 line compared to the healthy cell line, leading us to hypothesize that this receptor could also be involved in altered osteoblastogenesis in GSD. However, data concerning the involvement of GPR55 in bone metabolism are too few to fully understand the importance of this receptor in the onset and progression of skeletal diseases.

Most studies evaluating the possible mechanism of action of ES in bone remodeling have focused mainly on the action of CNR1 and CNR2 receptors and much less on the other components of the ES. In fact, there is also one work on the stimulation or inhibition of ES enzymes in bone cells, which reports that, for example, inhibition of FAAH would reduce osteoclast proliferation and differentiation and prevent bone loss [41].

A peculiar aspect of GSD is an excessive proliferation of blood and lymph vessels that tend to invade the eroded bone [1]. Some studies have reported that a high number of macrophage-like cells have been observed in the bone lesions of GSD patients. It has been hypothesized that these cells might not only represent the progenitors of osteoclasts, but that they might contribute to the secretion of factors such as Vascular Endothelial Growth factors (VEGFs) and consequently promote angiogenesis and lymphangiogenesis [4]. Given that macrophages express both CB1 and CB2 [42], the modulation of which would be able to regulate VEGF-A and angiopoietin (ANGPT) secretion [43], it would be interesting to understand whether ES could also play a role in this respect in the pathogenesis of GSD.

Therefore, our preliminary data showed for the first time that the ES is expressed in an in vitro cellular model of GSD, leading to the conclusion, on the basis of the collected data and on the basis of the studies mentioned above, that this complex network could play a role in the pathogenesis and progression of GSD.

Furthermore, in this study, we have also shown for the first time how the multipotency capacity of GSD cells was altered, representing the principal cause of the bone loss that characterizes this pathology.

Considering everything, however, our work has limitations. Mesenchymal stem cells from a single GSD patient were used, which did not allow us to uniquely correlate ES deregulation with the pathogenesis of the syndrome. Furthermore, the limited information we had about the patient did not allow us to understand whether any treatment carried out could have influenced the expression of the various ES components in the cells under investigation. Lastly, in addition to BMMSCs and OBs, other cellular actors contribute to the development of the syndrome, such as osteoclasts that resorb bone and endothelial cells that give rise to blood and lymphatic vessels; therefore, it would be interesting to investigate whether the expression of ES components could be altered and thus contribute positively to their proliferation and activity.

However, it must be kept in mind that GSD is an extremely rare skeletal disease with few patients in the world, from which tissues such as bone marrow are not even easily obtained. For this reason, we hope that our results will spur the scientific community on to explore this topic in their patients as well.

In conclusion, this is only a preliminary study, and further future analyses are needed to understand the role of the ES during osteogenic differentiation better and to try to comprehend what the molecular mechanisms involved in GSD pathogenesis are. In addition to this, the demonstration that the ES is present in our GSD in vitro model could pave the way to a study of the effects of the natural components of Cannabis Sativa as possible future new molecules that could be useful in the treatment of GSD and other bone diseases.

## 4. Materials and Methods

### 4.1. Primary hBMMSC Culture from GSD Patient

An hBMMSC primary culture was obtained from a human bone marrow sample collected from a Gorham-Stout patient from Prof. Brandi during her visiting years at the National Institutes of Health (NIH, Bethesda, MD, USA).

The bone marrow biopsy sample derived from the osteolytic area in the femur was immediately placed in McCOY’S 5A medium supplemented with 22 mM HEPES and 100 IU/mL penicillin, as well as 100 mg/mL streptomycin, pH 7.4.

The sample was minced into small fragments and subjected to enzymatic treatment in Ham’s F12 Coon’s modification medium supplemented with 20% fetal bovine serum (FBS), 100 IU/mL penicillin, 100 µg/mL streptomycin, and 3 mg/mL collagenase type II for 3 h at 37 °C. After that, a mechanical dispersion was performed.

The obtained cellular suspension was plated into a 100 mm Petri dish at 37 °C in a humidified atmosphere with 5% CO_2_ in DMEM/F12 medium supplemented with 10% fetal bovine serum (FBS), 100 IU/mL penicillin, and 100 mg/mL streptomycin (Growth Medium, GM). The medium was replaced with fresh GM every 3 days and once confluence was reached, the isolated cells were detached using Trypsin-EDTA and used for cell expansion, cryopreservation, and all the successive described experiments. The primary cell line was marked as hBMMSC-GS-1.

In this study, to evaluate the biological characteristics of hBMMSCs derived from a GSD patient, we used primary human-derived bone marrow mesenchymal stem cells from a healthy patient (hBMMSCs). An hBMMSC line was established by Prof. Brandi during her visiting years at NIH.

### 4.2. Immunofluorescence Staining

Immunofluorescent staining on isolated hBMMSC-GS-1 fixed in 4% PFA/DPBS was performed to investigate the presence of the mesenchymal stem cell (MSC) markers (i.e., CD44, CD90, and CD105) and Hematopoietic Stem Cell (HSC) markers (i.e., CD34), using primary antibodies.

After fixation, cells were permeabilized by 0.2% Triton X-100/DPBS at 37 °C in humidified air with 5% CO2. Cells were then washed three times with DPBS and treated with RNase diluted 1:100 with 2% BSA/DPBS. Cells were then washed three times and stained with primary antibodies (anti-CD44 (Invitrogen, Waltham, MA, USA), anti-CD90 (Abcam, Cambridge, UK), anti-CD105 (Invitrogen, Waltham, MA, USA), and anti-CD34 (Abcam, Cambridge, UK)).

After incubation in a humid microenvironment at 4 °C overnight, the primary antibody was removed, and cells were stained with the secondary antibody (goat anti-Mouse Alexa Fluor 635 IgG (H + L), (Life Technologies, Carlsbad, CA, USA)) in a dark humid environment at room temperature for 45 min. Cells were then washed several times by DPBS and counterstained for nuclei with propidium iodide staining (10–5 M in DPBS).

As a negative internal control, we used cells marked with only the secondary antibody. Stained cells were examined with 20× and 63× at room temperature on a Laser Scanning Confocal Microscope (LSM-900, ZEISS) (ZEISS, Oberkochen, Germany).

### 4.3. Adipogenic Differentiation

The hBMMSC-GS-1 line, once it reached 90–95% confluence in the Petri dish, was detached with Trypsin-EDTA and placed on a 24-well plate at a cell density of 1 × 10^4^ cells/cm^2^ in GM until reaching 80–90% confluence. At this point, the medium was changed to adipogenic medium (AM) consisting of DMEM/F-12 medium supplemented with 10% FBS, 100 IU/mL penicillin, 100 mg/mL streptomycin, 0.5 mM isobutylmethylxanthine (IBMX), 1 μM dexamethasone, and 1 μM bovine insulin. The AM was refreshed twice a week.

After 30 days, the cells in GM (control) and AM were colored by a freshly prepared Oil Red O working solution and lipidic vesicles were immediately observed through brightfield microscopy (LSM-900, ZEISS).

The adipogenic differentiation assay was also performed on the hBMMSC line isolated from the healthy donor to evaluate the adipogenic differentiation potential of both of the isolated hBMMSC lines.

### 4.4. Osteogenic Differentiation

hBMMSC lines were plated on 24-well plates at a cell density of 1 × 10^4^ cells/cm^2^ in GM until reaching 90% confluence. Afterward, the medium was changed with osteogenic medium (OM), composed as follows: DMEM/F12 medium supplemented with 10% FBS (origin South America, BioWhittaker), 100 IU/mL penicillin, 100 mg/mL streptomycin, 10 nM dexamethasone, 0.2 mM 2-phospho-ascorbate, and 10 mM β-glycerophosphate. The OM was refreshed twice a week, and the osteogenic phenotype was observed over a period of 4, 7, 14, 21, 28, and 35 days of osteogenic induction.

The osteogenic phenotype was evaluated through the dosage of enzyme activity of alkaline phosphatase (ALP) and hydroxyapatite (HA) deposition. Therefore, cells were washed twice with Dulbecco’s phosphate-buffered saline (DPBS), fixed in 4% PFA/DPBS, and washed three times with ultrapure distilled H2O.

For ALP cytochemical staining, the fixed cells were washed twice with DPBS, stained with a specific dye mixture solution freshly prepared and composed of 5 mg naphthol-AS-MX phosphate sodium salt in 1 ml of dimethyl sulfoxide (DMSO) and 40 mg Fast Red Violet LB placed in 50 mL of Tris-HCl Buffer, 280 mM, pH 9.0, and incubated for 30 min at 37 °C. The cytochemical staining for ALP was monitored every 10 min through the microscope. The ALP positive cells were stained in red, and nuclei, counterstained with methyl green, were observed through brightfield microscopy (LSM-900, ZEISS).

For HA cytochemical staining, Von Kossa staining, the HA deposits were stained with 1% nitrate solution in ultrapure distilled H2O and incubated under ultraviolet light for 2 h. After that, the unreacted silver solution was removed with 5% sodium thiosulfate solution for 5 min and washed several times with ultrapure distilled H_2_O. HA deposits and nuclei, counterstained in green with methyl green, were observed through brightfield microscopy (LSM-900, ZEISS).

We also quantified both the enzymatic activity of ALP and the amount of HA deposits produced in osteogenically induced hBMMSC-GS-1 and hBMMSC lines, respectively. ALP activity and HA quantity were measured with an LS55 spectrofluorometer (PerkinElmer). ALP activity was measured at 365 nm λ excitation and 445 nm λ emission and expressed in μU ALP/ng DNA using a standard curve of 4-methylumbelliferone (4-MU) 50 nm–10 μM in 280 mM Tris-HCl buffer pH 9.0.

For HA assay, since cells were grown in OM supplemented with 1 mg/mL Calcein, each well was incubated with 2 mL of 50 mM NaEDTA for 30 min at 37°; then, the fluorescence was measured with an LS55 spectrofluorometer (PerkinElmer) at 494 nm l excitation and 517 nm l emission and expressed in mg HA/ng DNA using a standard curve of HA 25 ng/mL–500 mg/mL solubilized in 50 mM NaEDTA. All tests for ALP activity and HA quantity were performed in quadruplicate.

### 4.5. Gene Expression Analysis of ES Components

The expression of ES components in hBMMSC-GS-1 and in hBMMSC lines was evaluated on cells cultured in GM by real-time qualitative polymerase chain reaction (RT-PCR) analyses. Total RNA was extracted using Qiazol Lysis Reagent (Qiagen) according to the manufacturer’s protocol. The concentration, purity, and integrity of the total RNA extracted were measured with an ND-1000 spectrophotometer (NanoDrop Technologies, Wilmington, DE, USA) and also with electrophoresis run on a 0.8% agarose gel. A total of 1 μg of total RNA was reverse-transcribed using TaqMan™ Reverse Transcription Reagent kit (Invitrogen™), according to the manufacturer’s protocol. To verify the correct reverse transcription reaction, qualitative PCR was performed using 1 μL cDNA as a template and 1 mM of each primer (forward and reverse) of the gene housekeeping Glyceraldehyde-3-phosphate dehydrogenase (GAPDH).

At this point, the RT-PCR reactions of Cannabinoid Receptor type 1 (CNR1), type 2 (CNR2), transient receptor potential cation channel subfamily V member 1 (TRPV1), G protein-coupled receptor 55 (GPR55), N-acyl phosphatidylethanolamine phospholipase D (NAPE-PLD), diacylglycerol lipase alpha (DAGLα), and fatty acid amide hydrolase (FAAH) genes were performed using GAPDH as the control. Reverse transcription products (1 μL) were amplified using a Nexus thermocycler (Eppendorf) using 25 mL reaction mixture containing 1 mM of each primer and HotStarTaq^®^ DNA Polymerase (Qiagen, Hilden, Germany) (Table 1), with a standard thermal profile.

At this point, genetic expression analysis by quantitative real-time PCR (qRT-PCR) was performed for each ES component gene, listed above, and GAPDH was used as the housekeeping gene, using Rotor Gene Q (Qiagen). Reactions were performed using TaqMan 5′-exonuclease assay, following the thermic profile according to manual instructions (GoTaq^®^ Probe qPCR Master Mix, Promega, WI, USA).

The primers and internal labeled oligonucleotide TaqMan probes for each gene (Table 1) were designed by IDT (Integrated DNA Technologies, Coralville, IA, USA). The cDNA samples used for the construction of standard curves for quantitative analysis were subjected to PCR amplification for each gene, and PCR products were analyzed by 0.8% agarose gel electrophoresis, visualized by Midori Green Advanced DNA staining (Nippon Genetics Europe, Düren, Germany), in the presence of 50 bp DNA Ladder (Invitrogen™, Waltham, MA, USA), and then we performed agarose gel elution using the QIAquick^®^ Gel Extraction Kit (Qiagen, Hilden, Germany). The standard curves were generated by assessing serial cDNA dilutions (10-fold dilution for 8 logarithms) and plotting fluorescence versus the Ct (threshold cycle) based on dRn (baseline corrected, reference dye-normalized fluorescence). All points for standard curves and unknown samples were performed in triplicate. Negative control tubes with water were included in each real-time PCR run to detect any carry-over contamination. Target gene expression was normalized to the Glyceraldehyde-3-phosphate Dehydrogenase gene (GAPDH).

### 4.6. Statistical Analysis

At least three independent experiments were conducted and the data obtained were expressed as mean ± S.D. As a first step, the normality of the replicates and the homoscedasticity of the groups were verified with the Lilliefors test and the Levene test, respectively. The exact total number (*n*) for each experiment is indicated in the relative legend. Statistical significance of the differences between the media was determined for ALP activity, HA deposits, and RT-qPCR assays by one-way ANOVA analysis and post hoc Bonferroni’s multiple comparison test with a predefined experiment-wise probability α_T_ = 0.05, using GraphPad Prism 9 software (GraphPad Software, San Diego, CA, USA). Values with *p* < 0.05 were considered statistically significant.

## Figures and Tables

**Table 1:** Primers and TaqMan probes used for RT-PCR and qRT-PCR analyses. List of the TaqMan probes and primers used in the ES components’ gene analysis.

Gene	Primer Sequences (5′-3′) and TaqMan Probes	Ta (°C)	Amplicon Size (bp)
CNR1	Forward CAA GCC TCT CTG GCA CTT T Reverse CTG GTG GTT GGG CCT ATT T Probe FTG CTG CCT AZA ATC CAC TCT GCA GGQ	56	100
CNR2	Forward CAA TGA GGG ACT TGG GAG AAA Reverse AAG GAG GCC TTG ACA ATT AGG Probe FTG GGA AGT CZA GGG TAT CAG ATG GGA Q	60	114
TRPV1	Forward AGC TAC ACG GAC AGC TAC TA Reverse GTC TGC TCC GTT CTC CAC Probe FAT CGC CAT CZG AGA GAC GCA ACA TQ	57	102
GPR55	Forward TCT GTT GGT GTT GTA GGA AGA A Reverse ATG GCC AGC AGG TTG AG Probe FAC CCT ACA GZT TTG CAG TCC ACA TCC Q	56	149
NAPE-PDL	Forward GAT CAC AGC AGT GTT CCA AGT Reverse TCC CAG CCA TGT GAC TCT TA Probe FAA GAA GCT GZG AGT GAG GGA AGC TGQ	60	129
FAAH	Forward GGT GCA GAA GTT ACA CAG TAG AG Reverse AGT CTC ACA GTC AGC CAG ATA Probe FCC TGG GAA GZT GAA CAA AGG GAC CAQ	64	124
DAGL	Forward GTG GAC ATC GTC TAT ACC TCC T Reverse CGG ATA CTG ATC ACC ACT TTC TT Probe FAT GAT GCG GZT CTA TGA AAC GCC CTQ	64	98
GAPDH	Forward AAT CCG TTG ACT CCG ACC TTC Reverse ACA GTA CAG CCG CAT CTT C Probe F CCA CAT CGC Z TCA GAC ACC ATG GGQ	69	179

Ta: annealing temperature; bp: base pair of amplicons; probes are marked with fluorochrome 6-carboxyfuorescein [6-FAM and Q quencher and fluorochrome Iowa Black FQ].

## Data Availability

The data presented in this study are available on request from the corresponding author.
